# Transcatheter Aortic Valve Implantation for Pure Aortic Regurgitation

**DOI:** 10.3390/jcm15093206

**Published:** 2026-04-22

**Authors:** Samuel Norman, Noman Ali, Daniel Blackman

**Affiliations:** 1 Department of Cardiology, Leeds General Infirmary, Leeds LS1 3EX, UK; 2 Leeds Institute of Cardiovascular and Metabolic Medicine, University of Leeds, Leeds LS2 9JT, UK

**Keywords:** structural heart disease, aortic regurgitation, transcatheter aortic valve implantation, JenaValve Trilogy, J-Valve

## Abstract

Transcatheter aortic valve implantation (TAVI) has transformed the management of severe aortic stenosis (AS), evolving from a therapy reserved for inoperable patients to a viable treatment across the spectrum of surgical risk. This success has stimulated innovation in transcatheter therapies for other valvular heart diseases, including aortic regurgitation (AR). In contrast to AS, AR is characterised by heterogeneous aetiologies, absence of annular calcification, larger and more elliptical annular dimensions, and concomitant aortopathy. These challenges have limited the efficacy and safety of conventional transcatheter aortic valves (TAVs), use of which in pure native AR is associated with high rates of valve embolisation, significant residual regurgitation, permanent pacemaker implantation, and mortality. The development of dedicated TAVs designed specifically for the treatment of AR has addressed many of these anatomical challenges. The JenaValve Trilogy and J-Valve systems incorporate leaflet-grasping mechanisms that enable secure anchoring independent of calcification, resulting in transformation of procedural and clinical outcomes. Recent prospective registry data, including the landmark ALIGN-AR trial, demonstrate high technical and procedural success rates, low residual regurgitation, acceptable safety profiles, and meaningful improvements in functional status and ventricular remodelling. These data have informed contemporary guideline updates, with the 2025 European Society of Cardiology (ESC)/European Association of Cardiothoracic Surgery (EACTS) Guidelines for the management of valvular heart disease issuing the first conditional recommendation for TAVI in selected patients with severe AR and the National Institute for Health and Care Excellence (NICE) recommending TAVI for native AR in patients for whom surgical AVR is not available or is high risk. This review summarises the clinical implications of AR, examines current guideline recommendations for management, and critically appraises the evidence supporting transcatheter treatment strategies.

## 1. Introduction

Initially developed as an alternative to surgical aortic valve replacement (SAVR) for patients with severe symptomatic aortic stenosis (AS) at prohibitive operative risk, TAVI has since been rigorously evaluated in numerous randomised clinical trials (RCTs) across the full spectrum of surgical risk [1,2,3,4,5,6]. This robust evidence base, combined with progressive streamlining of the procedure and advances in transcatheter aortic valve (TAV) design, has led to TAVI being the dominant mode of treatment for severe AS, exceeding the number of isolated SAVR in the UK since 2019 [7].

The remarkable success of TAVI in the treatment of severe AS has resulted in an explosion in transcatheter valve therapies over the last 20 years. However, the application of TAVI for the treatment of severe aortic regurgitation (AR) poses significant challenges. Severe AR typically results from aortopathy, congenital abnormalities or connective tissue diseases, whereas AS is overwhelmingly due to calcific degeneration. Conventional TAVs are designed to exploit the calcific nature of degenerative AS, with the TAV prosthesis anchoring to the calcified aortic valve leaflets. The absence of leaflet calcification in pure AR led to poor outcomes when traditional TAVs were used in this patient population. The development of dedicated TAVs, designed to overcome the anatomical challenges specific to AR, was necessary to drive sustained improvements in procedural success and clinical outcomes.

This review details the pathophysiology, natural history and epidemiology of AR and the development of dedicated TAVs and contemporary outcomes of TAVI in the treatment of severe AR, with a particular focus on current applications of TAVI in the UK. Literature cited in this review includes relevant recent evidence pertaining to TAVI for pure AR, with a particular focus on contemporary data, meta-analyses, and current national and international guidelines.

## 2. Aortic Regurgitation Overview

AR occurs when structural or functional abnormalities of the aortic valve or aortic root result in diastolic retrograde flow from the aorta into the left ventricle (LV). Unlike AS, which is predominantly calcific and degenerative, AR has a heterogeneous aetiology that may involve primary leaflet pathology or secondary annular and aortic root disease [8,9]. Primary valve disease typically produces eccentric regurgitant jets and includes age-related degeneration, bicuspid aortic valve, rheumatic heart disease, infective endocarditis, and inflammatory conditions such as systemic lupus erythematosus. Secondary AR is most commonly caused by chronic hypertensive aortopathy, with additional causes including connective tissue disorders, inflammatory aortitis, syphilis, and aortic dissection, usually resulting in central regurgitation.

Clinically significant VHD is common and increasingly prevalent with advancing age [10]. Population screening studies report clinically significant (moderate or severe) VHD in 11–15% of individuals aged ≥65 years. AR is the fourth most common VHD, with mild or greater disease present in approximately 10% of the general population and clinically significant AR affecting 0.7–1.6% in contemporary cohorts from the UK, USA, and China [11,12,13,14,15].

The natural history of AR is variable. Asymptomatic patients with preserved LV function may remain stable for years; however, once symptoms or LV dysfunction develop, prognosis declines significantly, with untreated symptomatic severe AR associated with a mortality of 15.4–24.9% at 2 years [16,17,18,19]. Despite this, intervention rates remain low, particularly among elderly patients and those with LV dysfunction [16,19,20,21,22], underscoring the need for improved recognition and expanded therapeutic options.

## 3. Transcatheter Valves for Aortic Regurgitation

**Figure 1:**
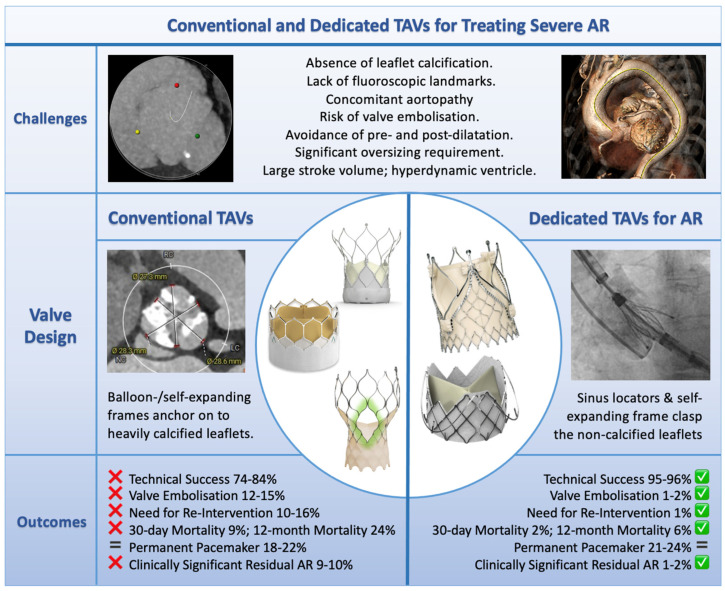
(Central illustration): transcatheter aortic valve implantation for severe native aortic regurgitation: why dedicated devices matter.

Conventional TAVs were designed for the treatment of degenerative calcific AS and are dependent on cusp calcification for anchoring. The absence of calcification in patients with pure AR makes anchoring ineffective, and as a consequence, valve migration and embolisation are common. While aggressive oversizing to mitigate this risk has been employed, this has led to an increased risk of annular or aortic injury [23,24]. Additional anatomical challenges associated with transcatheter treatment of pure AR include large annular dimensions, elliptical annular anatomy, and concomitant ascending aorta dilatation (Figure 1).

### Outcomes with Conventional TAVs in Pure AR

The first reports of TAVI in AR using the CoreValve and Sapien TAVs (Medtronic and Edwards Lifesciences, respectively) in the early 2010s proved that TAVI for pure AR was feasible [25,26,27]. However, subsequent case series and registry data reported poor technical and procedural success rates, in the range of 60–80% [28,29,30,31], and high rates of significant complications—far exceeding those seen with TAVI for severe AS. Key registry data pertaining to the transcatheter treatment of pure AR with conventional TAVs are summarised in Table 1. Valve embolisation and/or requirement for a second TAV was seen in 12.4–16.6% of patients, clinically significant residual AR in 9–10%, and 30-day all-cause mortality was 5–10.9% [27,28,30,31,32]. Permanent pacemaker (PPM) rates were also high, ranging from 18–22.6%, likely reflecting the requirement for significant TAV oversizing to prevent valve embolisation, a common practice when treating AR with conventional TAVs. Outcomes using conventional TAVs improve when the likelihood of annular anchoring is rigorously evaluated on the pre-procedural TAVI CT [33]; however, procedural success is still wanting even when patients with a low probability of adequate anchoring are excluded (91%). Consequently, the use of conventional TAVs in patients with AR is relatively uncommon; the PANTHEON registry, reporting international data on second-generation TAVIs between 2014 and 2022, found that only 1.4% of procedures were performed for native AR [30].

## 4. Dedicated Devices for Aortic Regurgitation

Two AR-specific or ‘dedicated’ TAVI systems have been developed to overcome the anatomical challenges presented by severe native AR: the JenaValve Trilogy (JenaValve Technology; Irvine, CA, USA) and the J-Valve (JC Medical, Inc., Burlingame, CA, USA and Suzhou, China). Both valve frames have incorporated ‘locators’ which engage the sinuses of Valsalva and grasp the aortic valve leaflets and a self-expanding frame which provides additional anchoring in the annulus and left ventricular outflow tract. Both valves require detailed pre-procedural planning using cardiac-gated multi-detector computed tomography (CT) to ensure patients have suitable vascular anatomy for valve delivery, an aortic annulus within the treatment size range, and an adequately sized ascending aorta to unsheathe the TAV locators on approach to the native valve. Table 2 details a comparison between the valve systems and contemporary conventional TAVs.

### 4.1. JenaValve

**Figure 2:**
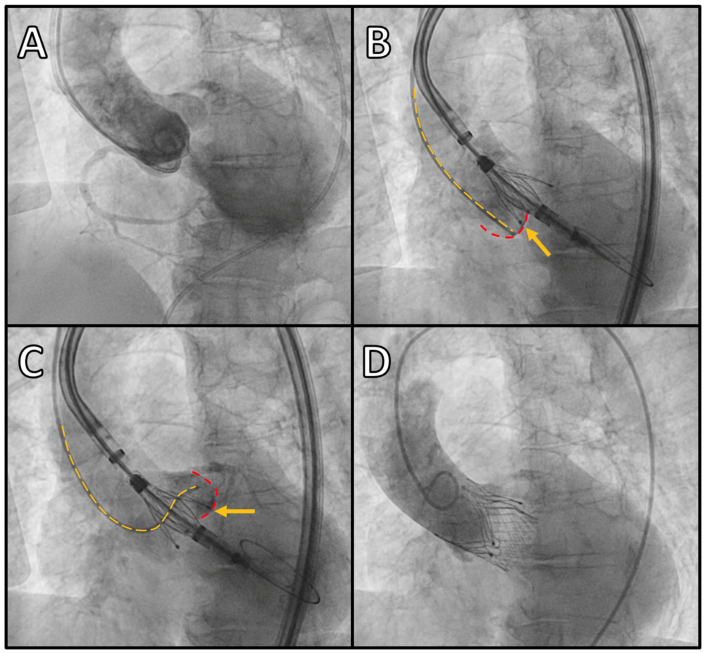
Example of JenaValve Trilogy deployment in a patient with severe AR. (**A**) Aortogram performed in the three-cusp (co-planar) view with complete opacification of the LV reflecting severe AR. (**B**,**C**): Selective contrast injections in the NCC (panel (**B**)) and LCC (panel (**C**)) using MP and AL1 catheters, respectively, to ensure the valve locators are positioned within the cusp ahead of deployment. Yellow dotted lines trace the catheter position; red dotted lines trace the base of the relevant sinus; yellow arrows identify the valve locators engaged within the sinus. (**D**): Aortogram post-valve deployment with no residual AR. AL1: Amplatz Left 1 catheter; AR: aortic regurgitation; LCC: left coronary cusp; LV: left ventricle; MP: multi-purpose; NCC: non-coronary cusp.

The Trilogy valve consists of a supra-annular porcine pericardial valve and a nitinol self-expanding frame. The three locators engage the aortic sinuses and then clip onto the native valve leaflets using a rapid-deployment self-expanding mechanism. The locators can be rotated within the sinus of Valsalva to ensure a central position in each sinus. This mechanism anchors the frame to the three leaflets, ensuring device stability and maintaining commissural alignment (Figure 2).

Overview of the challenges faced in treating severe AR with transcatheter therapies (top panel), current dedicated and conventional treatment options available (central circle) and outcomes with contemporary dedicated and conventional devices (bottom panels; data for conventional TAVs is from Yoon et al. [29] and the PANTHEON registry [30]; data for dedicated TAVs comes from the ALIGN-AR pivotal trial and continued access registry [35,37] and the Gao et al. J-Valve meta-analysis [36]. ‘Technical Success’ refers to the Valve Academic Research Consortium (VARC)-3 definition). Valves depicted in the central circle (clockwise from 12 o’clock): JenaValve Trilogy, J-Valve, Evolut FX+, SAPIEN 3 Ultra Resilia, and Navitor Vision. AR: aortic regurgitation; TAVI: transcatheter aortic valve implantation; TAV: transcatheter heart valve.

The first iteration of the JenaValve system was delivered anterogradely via a trans-apical (TA) approach and was European Conformity (CE) approved in 2013 for the treatment of severe AR. Early experiences with the TA system were encouraging, with favourable rates of procedural success and low rates of residual AR [38], but apical complications and a concerning 30-day mortality rate led to JenaValve withdrawing the valve in 2016. The system was redesigned and modified to be delivered retrogradely via a trans-femoral (TF) approach, and the second-generation device received CE approval for AR and AS in 2021. The new TF system, rebranded as JenaValve Trilogy, uses an 18-French delivery system, which allows the valve to be rotated within the ascending aorta in order to facilitate cusp engagement, and an external sealing ring that conforms to the annulus to prevent paravalvular leak. The valve is available in three sizes (small [23 mm], medium [25 mm] and large [27 mm]), which allows treatment of aortic annulus perimeters of 66–90 mm. The delivery system is lubricious and flexible, allowing treatment of patients with challenging vascular access or aortic anatomy, including via alternative access routes [39].

Early registry data demonstrated encouraging outcomes with the JenaValve Trilogy, with near-universal procedural and technical success and favourable clinical outcomes [40]. The landmark ALIGN-AR trial further established the efficacy of the Trilogy device in the treatment of AR [35]. This single-arm, multi-centre study prospectively evaluated the safety and efficacy of the JenaValve Trilogy valve in 180 patients with moderate-to-severe or severe AR at high surgical risk. The mean patient age was 75.5 years. Of note, the mean STS-PROM score was 4.1%, corresponding to an intermediate surgical risk population; however, most patients (89%) were considered high risk by virtue of comorbidities that are not captured by the STS-PROM framework, particularly frailty.

Comparators for the safety and efficacy endpoints were derived from contemporary TAVI studies treating AS in high-risk populations. ALIGN-AR met all non-inferiority endpoints with high rates of technical (95%) and procedural success (92.8%), coupled with low rates of procedural and early complications (no procedural deaths, 30-day mortality 2%, disabling stroke 1%, non-disabling stroke 1%, and clinically significant PVL 0.6%). The primary efficacy endpoint of mortality at 1 year was 7.8%, meeting the pre-specified non-inferiority threshold. The only concern highlighted in this study was the relatively high rate of PPM implantation following TAVI (24%). The ALIGN-AR authors note improvement in the PPM implantation rate over the study period, thought to be due to modification of the valve sizing matrix and implantation techniques. However, relatively high PPM implantation rates have also been observed in non-randomised data [41]. This is likely to be due, at least in part, to the underlying disease process rather than the treatment, as annular ectasia is more frequent in patients with AR, and the resultant mechanical stretch can impact conduction through the bundle of His. Support for this assertion comes from the observation that PPM implantation rates are also significantly higher following SAVR when performed for AR rather than AS [42,43].

The two year-outcomes of ALIGN-AR were published in late 2025 [37] and included 700 patients, 180 from the pivotal cohort and 520 from the continued access registry. Primary safety and efficacy endpoints again met prespecified performance goals, and rates of significant complications were low (1.6% valve embolisation, 1.4% 30-day mortality). Imaging endpoints, including LV mass index and end-systolic volume index, and patient symptoms as evaluated using New York Heart Association and Kansas City Cardiomyopathy Questionnaire scores, were all significantly improved from baseline.

JenaValve Trilogy is being implanted across the UK, Europe and China, while regulatory approval for JenaValve Trilogy in the US remains pending. The first RCT comparing JenaValve Trilogy to SAVR (Aortic Regurgitation Trial Investigating Surgery Versus Trilogy; ARTIST) is currently enrolling (NCT06608823). This trial will randomise patients with a clinical indication for AV intervention, who are not at high surgical risk, and who are appropriate for either therapy. The primary endpoint is a composite of all-cause mortality, stroke, and unplanned cardiac rehospitalisation at 12 months.

### 4.2. J-Valve

The J-Valve is a bovine pericardial valve seated within a nitinol frame with an external polyester sealing skirt. Similar to the JenaValve locators, the frame has three U-shaped external anchors that engage the aortic sinuses and grasp the leaflets on valve release, securing the valve to the native anatomy and creating a seal at the level of the annulus. Of note, J-Valve is able to treat an annular perimeter up to 104 mm, significantly larger than the 90 mm upper limit of JenaValve Trilogy.

The development of J-Valve follows a similar timeline to JenaValve; the first iteration required a TA approach and, despite initial successes with impressive procedural success rates [44,45], the challenges associated with TA delivery resulted in the company overhauling the design and re-introducing the device as a TF system. The contemporary valve is available in five sizes (22, 25, 28, 31, and 34 mm) and can be delivered from either a TA or TF approach.

North American registry data have shown satisfactory procedural success rates (81% overall, but with improvements over time indicative of a learning curve effect) and low complication rates [46]. A recent meta-analysis from China that included data from nine studies totalling 552 patients reported a procedural success rate of 96% and in-hospital and 30-day mortality rates of 3% [36]. Interestingly, the PPM rate was quite low at 7%, though the 33% incidence of mild or moderate paravalvular leak (PVL) is notable. As with JenaValve, whilst these non-randomised data are encouraging, RCT data are still awaited.

The J-Valve is approved for use in China and on compassionate grounds in the USA and Canada but is not currently available in Europe. A TF single-arm study (J-Valve Transfemoral Pivotal Study (JOURNEY)) is currently enrolling patients in the USA, Canada, Europe and Japan (NCT06455787).

### 4.3. JenaValve vs. J-Valve Comparisons

There have been no randomised or head-to-head comparisons of the two AR-dedicated TAVs. However, a meta-analysis of non-randomised, predominantly retrospective data sets suggests that PPM implantation at 30 days is more common following treatment with JenaValve (21% vs. 6%; *p* < 0.01), with a trade-off of higher rates of moderate or severe residual AR with J-Valve (1% vs. 3%; *p* < 0.01) [47]. No other differences between the valve platforms have been identified.

## 5. Comparison Between Dedicated vs. Conventional Percutaneous Devices in AR

While there are no RCTs comparing dedicated and conventional TAVs for the treatment of pure severe AR, large meta-analyses of non-randomised data have comprehensively demonstrated superior outcomes and lower rates of significant complications when TAVI is performed using dedicated devices. The largest meta-analysis to date, published in 2025 and including 34 studies (6 prospective, 28 retrospective) and 2162 patients (1193 treated with dedicated devices and 969 with conventional devices), identified significantly better outcomes in patients treated with dedicated devices, including procedural success (93% for dedicated devices vs. 82% for conventional devices; *p* < 0.01), need for reintervention (4% vs. 10%; *p* < 0.01), 30-day all-cause mortality (3% vs. 9%; *p* < 0.01) and 1-year all-cause mortality (6% vs. 24%; *p* < 0.01) [47]. Procedural complications were less frequent in the pooled dedicated devices group, including valve embolisation (2% vs. 8%; *p* < 0.01), requirement for PPM (11% vs. 20%; *p* < 0.01), major bleeding (3% vs. 7%; *p* < 0.01), and clinically significant residual AR (4% vs. 10%; *p* = 0.03). There were no differences between the groups in terms of stroke risk or major vascular complications [47].

## 6. Guidelines for the Management of Aortic Regurgitation

The ESC/EACTS and American College of Cardiology/American Heart Association (ACC/AHA) guidelines both uphold the central role of timely surgery in the treatment of severe AR with Class I recommendations [48,49]. In patients with severe AR who are candidates for surgery, both guidelines endorse the use of tailored decision-making, where factors such as patient age, modified Carpentier classification, concomitant aortopathy and local expertise inform treatment selection, and the use of the Heart Team and Comprehensive Valve Centres in complex decision-making.

The most recent iteration of the ESC/EACTS VHD guidelines, published in August 2025, was the first to support the use of TAVI in the treatment of severe AR in patients who are ineligible for surgery, receiving a class IIb recommendation [49]. Earlier ESC/EACTS VHD guidelines had cautioned that TAVI should only be considered in select high-risk or inoperable AR patients in high-volume centres, citing the lack of randomised evidence to support wider use. The new guidelines also support the use of dedicated valves in this setting, citing the increased risk for valve migration and residual significant AR with conventional devices. The most recent ACC/AHA VHD guideline was released in 2020, prior to the publication of notable relevant publications. As such, the only American guideline recommendation pertaining to TAVI is a Class III recommendation cautioning risk of harm [48]. An updated VHD guideline from the ACC/AHA is anticipated in late 2026; endorsement of TAVI for the treatment of some AR patients is expected.

## 7. TAVI in the United Kingdom

All TAVI procedures performed in the UK are captured by the National Institute for Cardiovascular Outcomes Research (NICOR) TAVI Registry, which publishes annual demographic, procedural, outcomes and quality data. The most recent report included data from 10,367 TAVI cases performed across 2024–2025 from 31 NHS TAVI hospitals across England, Wales and Northern Ireland [7]. Unfortunately, while the indication for TAVI is routinely recorded in the NICOR dataset, these data are not included in the annual report, so the proportion of patients undergoing TAVI for severe AR in the UK and the outcomes data for this patient cohort is not available.

The UK National Institute for Health and Care Excellence (NICE) procedural guidelines pertaining to TAVI for AR (HTG752; previously IPG805) were published in 2025 and recommend that TAVI may be considered for patients with native AR who are at high or prohibitive surgical risk or who are unsuitable for SAVR. In patients who are suitable for SAVR or who are not at high surgical risk, TAVI should only be performed in the context of formal research studies. Across all cases, NICE emphasises the importance of shared decision-making, the multidisciplinary heart team, involvement of experienced centres, and mandatory reporting of outcomes. Broader adoption is contingent on future trials demonstrating satisfactory safety, efficacy and durability outcomes.

## 8. Limitations and Future Directions

### 8.1. Lack of Data

All data pertaining to dedicated AR devices are non-randomised; randomised data are needed to definitively establish the efficacy and safety of these devices in the patients eligible for, but at high surgical risk for, SAVR. There are also few data on outcomes in patients at low or intermediate surgical risk; SAVR remains the gold standard in this patient population. Similarly, there remains a need for long-term durability data, as well as more data pertaining to PPM implantation rates and the feasibility of coronary re-access, before use of these devices can be expanded into younger and lower-risk populations. Development of updated valve sizing matrices for pure AR and optimisation of dedicated device implantation techniques to reduce PPM implantation rates should be active areas of future enquiry.

### 8.2. Limitations of Valve Design

JenaValve and J-Valve are both designed to treat tricuspid aortic valves; patients with bicuspid anatomy were excluded from the ALIGN-AR trial and other early investigative trials. Since the anchoring mechanism of both valves depends upon grasping three leaflets, the treatment of patients with severe AR in the setting of bicuspid valves will remain challenging with existing technology. Advances in this area may require exploration of off-label use of JenaValve or J-Valve, or the development of second-generation devices capable of treating non-tricuspid anatomy.

Concomitant aortopathy and annular dilatation are significant challenges in this patient population. In the ALIGN-AR pivotal trial, 47.9% of screened patients were found to be ineligible, and roughly half of these were due to anatomical issues relating to the annulus or aorta: annuli outside the sizing range (36 of 346 screened; 10.4%), horizontal aorta (35; 10.1%), and aortic dilatation > 50 mm (6; 1.7%) [35]. A larger JenaValve size is in development, but the presence of significant annular dilatation will continue to represent a treatment challenge that may render potential patients untreatable. As mentioned, the J-Valve is able to treat larger annuli than the JenaValve but is currently unavailable in Europe. Future iterations of both dedicated valve platforms should make design changes to overcome the anatomical challenges presented by AR with associated aortopathy.

The use of CT planning to evaluate which patients have a higher chance of successful TAV anchoring has increased the rates of procedural success and reduced the rates of complications, particularly PPM requirements, when using conventional TAVs in pure AR [33]; future studies should further evaluate the use of multi-modal imaging to plan and guide TAVI procedures with dedicated TAVs.

## 9. Conclusions

The emergence of dedicated TAVs for the treatment of pure AR is a major step forward in the management of this increasingly prevalent disease. Randomised data and longer-term follow-up are needed to address questions of durability, patient selection, and relative effectiveness compared with surgery. As experience grows and devices continue to iterate, TAVI looks certain to assume an increasingly important role in the management of pure AR.

## Figures and Tables

**Table 1:** Key trials and registries evaluating outcomes following transcatheter treatment of pure aortic regurgitation using conventional and dedicated platforms.

Study	Design; TAVs Studied	N	Technical Success	Mortality	Valve Embolisation	Second Valve in Index Procedure	Residual AR (≥Moderate)	PPM Implantation
**Yoon et al. 2017** [29]	Retrospective registry. Mostly conventional TAVs (CoreValve 33.2%, Evolut R 15.1%, Sapien 3 12.4%); 19.3% JenaValve	331	74.3%	30-day: 10.9% 1-year all-cause: 24.1% 1-year CV: 15.6%	Not reported	16.6%	9.6%	18.2%
**Poletti et al. 2023** [30] PANTHEON Registry	Retrospective registry. Various conventional TAVs (Evolut Pro/R 37.8%, MyVal 19.9%, Sapien 3/Ultra 14.4%)	201	83.6%	In-hospital: 5% 1-year all cause: 11%	12.4%	10.4%	9.5%	22.3%
**Poletti et al. 2024** [34] PURPOSE Registry	Retrospective registry. 66% conventional TAVs; Jena Valve 33%	256	Conventional: 81% JV: 98%	Conventional in-hospital: 6.6% JV in-hospital: 1.1%	Conventional: 15% JV: 1.1%	Conventional: 11% JV: 1.1%	Conventional: 10% JV: 1.1%	Conventional: 22% JV: 24%
**Vahl et al. 2024** [35] ALIGN-AR Pivotal Trial	Prospective, single-arm study. Jena-Valve Pitoval Trial.	180	95%	30-day: 2% 1-year all-cause: 7.8%	2.2%	1.1%	0.6%	24%
**Gao et al. 2025** [36] J-Valve Meta-analysis	Meta-analysis of nine J-Valve single-arm trials (five retrospective, four prospective)	552	96%	In-hospital: 3% 30-day: 3% 1-year all cause: 6%	0.2%	0.2%	2.5%	6.7%
**Makkar et al. 2025** [37] ALIGN-AR Continued Access Registry	Prospective, single-arm registry following on from the ALIGN-AR Pivotal trial	700	95%	30-day: 1.6% 1-year all-cause: 7.7% 2-year all-cause: 13.3%	1.3%	0.7%	30-day: 0.5% 1-year: 1% 2-year: 1%	30-day: 21.6% 1-year: 27.2% 2-year: 27.7%

AR: aortic regurgitation; JV: Jena Valve; N = number of patients; PPM: permanent pacemaker; TAVs: transcatheter aortic valves.

**Table 2:** Overview of dedicated and conventional transcatheter heart valves.

	Name Manufacturer	Valve Design	Annulus Diameter Treatment Range (mm)	Annulus Perimeter (mm) or Area (mm^2^) Range
**Conventional TAVs**				
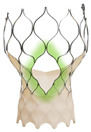	**Evolut Fx+** Medtronic	Supra-annular Self-expandable Recapturable Nitinol frame with porcine pericardium Four sizes available	18–30	56.5–94.2 mm
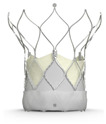	**Navitor Vision** Abbott Vascular	Intra-annular Self-expandable Recapturable Nitinol frame with bovine pericardium Five sizes available	19–30	60–95 mm
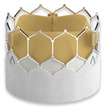	**SAPIEN 3 Ultra Resilia** Edwards Lifesciences	Intra-annular Balloon-expandable Non-recapturable Cobalt–chromium frame with bovine pericardium Four sizes available	18.6–29.5	273–683 mm^2^
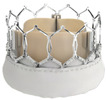	**MyVal Octacor** Meril Life Sciences	Intra-annular Balloon-expandable Non-recapturable Cobalt–nickel frame with bovine pericardium Nine sizes available	18.5–32.7	270–840 mm^2^
**Dedicated TAVs for Pure Aortic Regurgitation**				
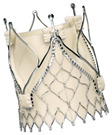	**JenaValve Trilogy** JenaValve Technology	Supra-annular Self-expanding Non-recapturable Nitinol frame with porcine pericardium Three sizes available	21–28.6	66–90 mm
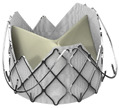	**J-Valve** JC Medical	Supra-annular Self-expanding Non-recapturable Nitinol frame with bovine pericardium Five sizes available	18–33	57–104 mm

## Data Availability

No new data were created or analysed in this study.
